# Virgin Olive Oil By-Products: Biological Activity of Phenolic Extract of Pâté on AGS Gastric Cells

**DOI:** 10.3390/ijms24097959

**Published:** 2023-04-27

**Authors:** Paola Faraoni, Lorenzo Cecchi, Maria Bellumori, Alessio Gnerucci, Francesco Ranaldi, Nadia Mulinacci

**Affiliations:** 1 Department of Experimental and Clinic Biomedical Sciences “Mario Serio”, University of Florence, Viale Pieraccini 6, 50139 Florence, Florence, Italy; francesco.ranaldi@unifi.it; 2 Department of Agricultural, Food and Forestry Systems Management (DAGRI), University of Florence, Piazzale Delle Cascine 16, 50144 Florence, Florence, Italy; lo.cecchi@unifi.it; 3 Department of NEUROFARBA, Division of Pharmaceutical and Nutraceutical Sciences, University of Florence, Via U. Schiff 6, 50019 Sesto Fiorentino, Florence, Italy; maria.bellumori@unifi.it (M.B.); nadia.mulinacci@unifi.it (N.M.); 4 Department of Physics and Astronomy, University of Florence, Via Sansone, 1, 50019 Sesto Fiorentino, Florence, Italy

**Keywords:** hydroxytyrosol, extra virgin olive oil, olive oil by-product, polyphenols, bioactive compound, AGS cells, antioxidant action, metabolism, enzymatic activities

## Abstract

Pâté is a by-product of olive oil production which represents an abundant source of phenolic compounds and can be used for food formulation, reducing its environmental impact and promoting a circular economy. In this context, the effects of a hydroalcoholic extract of pâté were evaluated for the first time in an AGS human cell line commonly used as model of gastric mucosa. Pâté was obtained from Tuscan olives; the total phenolic content was 16.6 mg/g dried extract, with verbascoside and secoiridoid derivatives as the most abundant phenols. The phenolic pâté extract did not alter viability, distribution of cell cycle phases or proliferation and migration of AGS cells at the tested concentrations. Seven enzymes were chosen to investigate the metabolic effect of the pâté extract in the context of oxidative stress. Pâté produced a statistically significant increase in the activity of key enzymes of some metabolic pathways: Lactate dehydrogenase, Enolase, Pyruvate kinase, Glucose 6-phosphate dehydrogenase, Citrate synthase, 3-Hydroxyacyl-CoA dehydrogenase and Hexokinase. Pre-treatments with the extract of pâté at 100 µg/mL or 200 µg/mL, as observed through PCA analysis, appeared able to counteract the enzymatic activity alterations due to oxidative stress induced by H_2_O_2_ 1 mM and 2 mM. The results indicate that dried pâté, due to its phenolic components, can be proposed as a new functional food ingredient.

## 1. Introduction

Currently, agricultural waste represents an important source of molecules with potential beneficial effects, and the possibility of recovering these molecules fits perfectly with the concept of the circular economy in agricultural production [1,2]. The supply chain of production of virgin olive oils from the olive fruit is an example of the production of high quantities of by-products, because the virgin olive oil yields are low (i.e., 12–20% of the fruit weight). Extra virgin olive oil (EVOO) contains high amounts of bioactive phenols, which can guarantee the recommended daily intake of phenols suggested by the EFSA in 2011: at least 5 mg/20 mL of oil per day to have protective cardiovascular effects [3]. One aspect to underline is that the great majority of phenolic compounds are lost during the olive milling process, and no more than 0.5% of the phenols contained in the intact fruit is transferred to the virgin olive oil [4]. Olive pomace, the main by-product recovered during virgin olive oil production by two-phase decanters [5], can be seen as a pollutant for the environment, but at the same time it is a valuable source of phenolic compounds [6]. Pâté is a destoned olive pomace that is recovered during, and not after, the separation of oil into the two-phase decanter and a Leopard^®^ system (Pieralisi Group S.p.A., Jesi, Italy) that protects phenolic compounds from oxidation. Dried pâté is a wet homogeneous olive pulp free from residuals of stone and husk, with a very good chemical stability when stored at room temperature under a vacuum. It was observed that 1 g of dried product can contain a phenolic total equivalent to that present in 200 mL of EVOO and a total phenols content from 50 to 150 g/kg of dried pâté. Notably, the phenolic molecules in the dried product, as well as its antioxidant power, remained stable when the product was subjected to irradiation [7]; furthermore, after an accelerated shelf-life test, only 16% of the phenols was lost [8]. For these reasons, many uses have been proposed in the cosmetic and pharmaceutical fields for its antioxidant activity [9]. By introducing pâté into the diet, a significant enrichment in phenolic compounds could be obtained to improve the health status by counteracting the free radicals involved in ageing and by regulating the metabolism.

Concerning the bioactivities of pâté, it was able to exert anti-aging effects on a model of human fibroblasts [6], it showed positive effects on the human microbiota when tested with SHIME^®^, an advanced in vitro gastrosimulator model [10], and it also exerted protective effects at tissue level on an animal model of intestinal bowel diseases [11]. Finally, pâté has already been used as a food ingredient of staple foods for human and animal consumption [12,13,14] and as a food supplement in tablet form [15].

AGS cells are widely chosen as a model to study the effects of certain food components at the gastric level prior to absorption in the small intestine. In particular, this cell model responds to oxidative stress in a manner comparable to that of non-tumour cells [16,17,18,19]. The effects of bioactive compounds on this cellular model have been poorly investigated. Indeed, very few studies can be found in the literature in this regard [20,21,22].

The aim of this work was to investigate the effects of a hydroalcoholic dried pâté extract containing a pool of phenols on the proliferation, migration and redox status of the gastric AGS cell line. Subsequently, the possible use of this phytocomplex was investigated as a countermeasure to the alterations of enzymatic activities due to oxidative stress induced by H_2_O_2_. The activities of seven key enzymes of some of the main cellular metabolic pathways (glucose phosphorylation and its entrapment in the cell, glycolysis, citoplasmatic NADH re-oxidation, pentose phosphate shunt, Krebs cycle and beta oxidation) were determined in cells pre-treated with two different concentrations of pâté and then subjected to oxidative stress by H_2_O_2_.

## 2. Results and Discussion

### 2.1. Chemical Composition of Pâté

**Figure 1:**
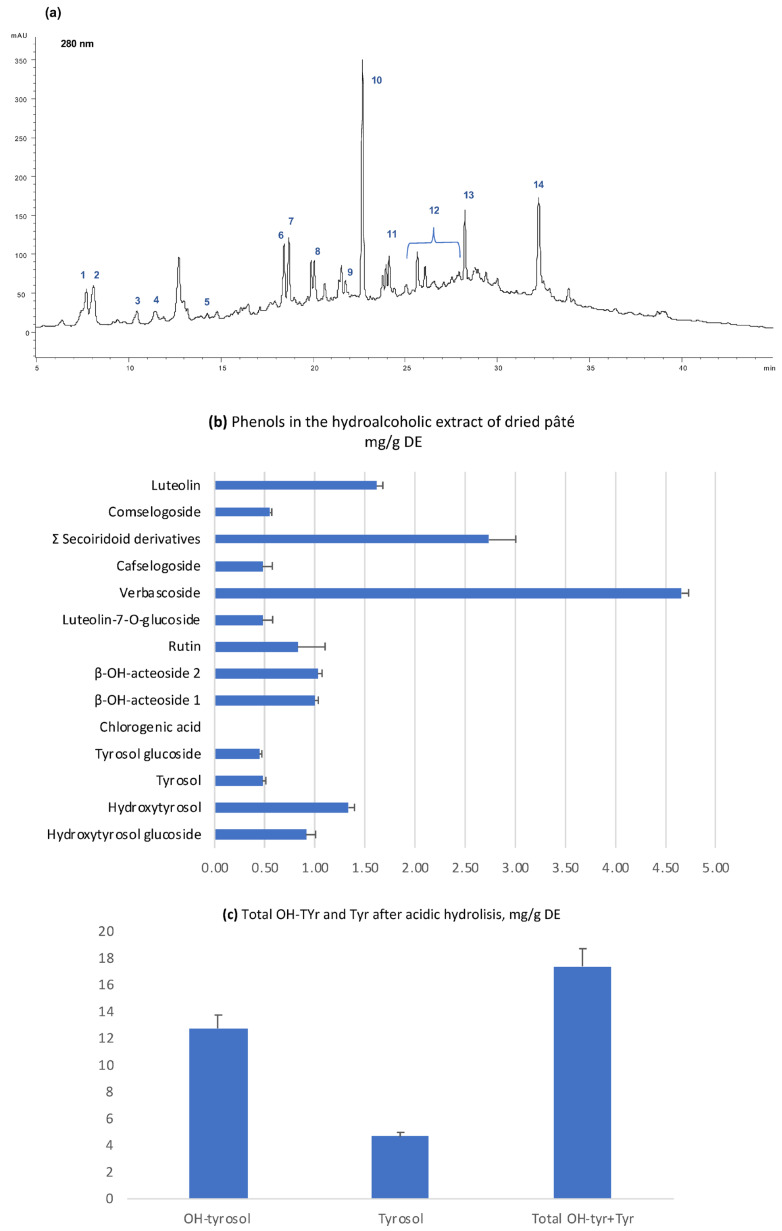
(**a**) Chromatographic profile of the hydroalcoholic extract of freeze-dried pâté: 1, hydroxytyrosol glucoside; 2, hydroxytyrosol; 3, tyrosol glucoside; 4, tyrosol; 5, chlorogenic acid; 6, β-OH-acteoside isomer 1; 7, β-OH-acteoside isomer 2; 8, rutin; 9, luteolin-7-*O*-glucoside; 10, verbascoside; 11, cafselogoside; 12, secoiridoid derivatives; 13, comselogoside; 14, luteolin. (**b**) Amount of each phenolic compound in the hydroalcoholic extract of freeze-dried pâté expressed as mg/g dried extract; (**c**) total content of tyrosol and hydroxytyrosol free and linked forms determined after acidic hydrolysis and expressed as mg/g DE.

The chemical composition was evaluated on the basis of the chromatographic profile of the hydroalcoholic pâté extract (Figure 1a). The profile of the pâté sample analysed in this study is in agreement with the phenolic content in the fruit of the olives and the enzymatic transformations that occur during the milling process which determine the disappearance of the oleuropein and its transformation into non-glycosylated secoiridoid derivatives. The pâté by-product, unlike EVOO, also contains numerous glycosylated forms including verbascoside and its hydroxylated derivatives (i.e., β-OH acteoside isomers 1 and 2). Using HPLC-DAD-MS, it was possible to define the relative content of each compound, including hydroxytyrosol and tyrosol, both in free and glycosylated forms, but also some glycosylated flavonoids such as rutin and luteolin-7-*O*-glucoside. Among the phenolic pool, secoiridoidic derivatives and verbascoside with its hydroxylated derivatives (β-OH-acteoside 1 and 2) were the main components of the extract (Figure 1a,b). The total amount of the phenolic compounds was 16.6 mg/g of dried extract, corresponding to 5.67 mg/g total phenols in the dried pâté.

Because several molecules in the extracts contain in their structure the group of hydroxytyrosol (e.g., secoiridoidic derivatives, verbascoside and its isomers), a simple phenol well known for its biological properties as also underlined by EFSA [3], a further determination was carried out by hydrolyzing the phenolic extract in acidic conditions to evaluate the sum of free and linked forms of tyrosol and hydroxytyrosol (Figure 1c). The total hydroxytyrosol content reached 12.7 mg/g on the dried hydroalcoholic extract of pâté (corresponding to 4.4 mg/g of dried pâté), almost three times higher than that of tyrosol, which reached 4.6 mg/g of dried extract of pâté. The in vitro antioxidant capacity of this latter phenol is lower than that of hydroxytyrosol.

The phytocomplex that characterized the hydroalcoholic extract of pâté was a mixture of phenolic compounds with different structures and different MW that also includes simple phenols and flavonoids. Overall, most of the molecules are characterized by the presence of a catecholic group in their structures, which is recognized as crucial to exerting antioxidant properties. The data in Figure 1c, which shows the prevalence of hydroxytyrosol on tyrosol, are in agreement with this latter consideration. Furthermore, it can be highlighted that approximately 1.5 g of dried pâté was larger than the minimum amount of total hydroxytyrosol requested by EFSA (5 mg) for applying a health claim to EVOO phenols [3].

### 2.2. Viability Assay with MTT Test and Cell Proliferation with Cytofluorimetric Analysis

**Figure 2:**
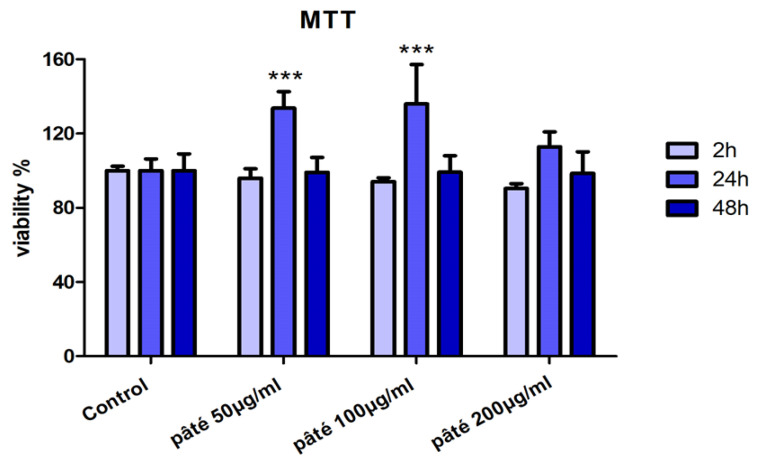
Cell viability after treatment with the hydroalcoholic extract of pâté at 50, 100 and 200 µg/mL for 2, 24 and 48 h by MTT assay. *** indicates statistically significant difference respect to the control at the same time (Student’s *t*-test *p* < 0.001).

The assessment of cell viability after incubation with the hydroalcoholic extract of pâté showed, for the treated samples with respect to control ones, a very slight reduction at 2 h, a statistically significant increase at 24 h (*p* < 0.001) for cells treated with 50 and 100 µg/mL, and levels comparable to those of control cells at 48 h for all the three tested concentrations. These observations indicate that at the concentration range of 50–200 µg/mL, the hydroalcoholic extract pâté is not cytotoxic (Figure 2).

**Figure 3:**
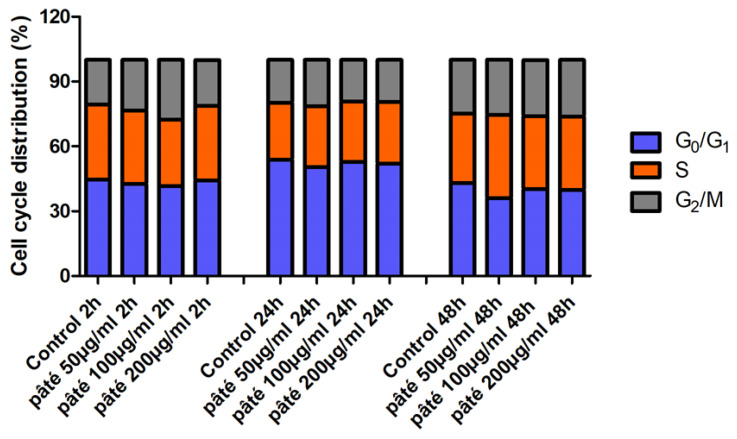
Percentage distribution in the different phases of cell cycle of samples treated with 50, 100 and 200 µg/mL of the extract of pâté 2 h, 24 h and 48 h.

The cytofluorimetric analysis of the cell cycle phase distribution in samples treated with 50, 100 or 200 µg/mL of the hydroalcoholic extract of pâté for 2, 24 and 48 h is similar to that of control samples during the observation time (Figure 3). These data suggest that, at the investigated concentrations, the treatment does not alter the proliferation of the AGS cells. On the basis of the results shown in Figure 2 and Figure 3, for the subsequent biological analyses, the intermediate and the higher concentrations of 100 µg/mL and 200 µg/mL were chosen.

### 2.3. Scratch Test Results

**Figure 4:**
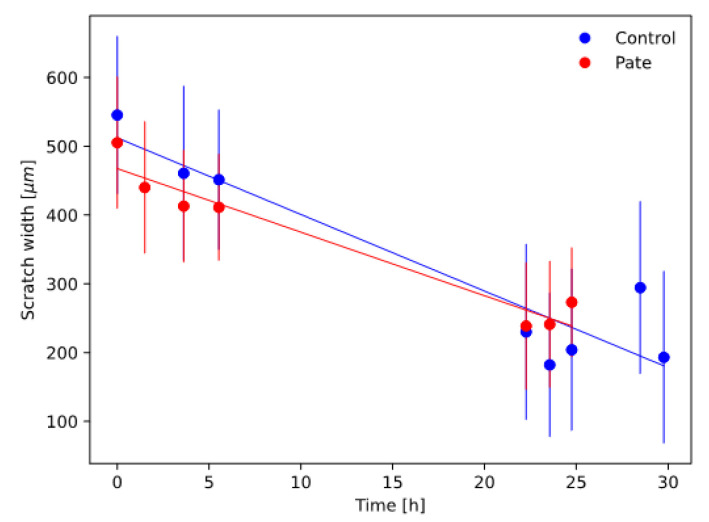
Scratch closure curves (coloured circles, with standard deviation error bars) and relative best-fit linear models (continuous lines).

In Figure 4, the measured scratch closure curves for the investigated samples are shown together with the best-fit linear models. Table 1 reports the measured closure velocity and closure time for the investigated samples.

Scratch assay closure modelling results showed that the pâté extract had a scratch closure velocity and closure time statistically similar to the control. This indicates that pâté did not significantly influence the cellular migration and proliferation with respect to the control.

### 2.4. Evaluation of ROS Production

**Figure 5:**
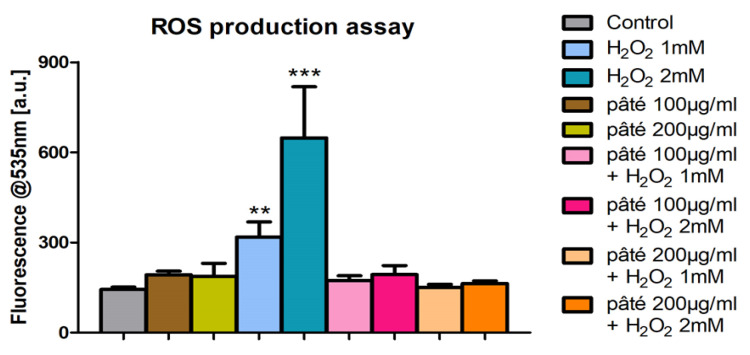
ROS assessment by the DCF-DA test in cells treated with the extract of pâté for 24 h and then incubated with two different concentrations of H_2_O_2_, 1 mM or 2 mM for 1 h. ** or *** indicates a statistically significant difference compared to the control sample (Student’s *t*-test ** *p* < 0.01, *** *p* < 0.001).

The evaluation of ROS production with the DCF-DA assay shows that the treatments with two concentrations of pâté (100 and 200 μg/mL) produced ROS levels statistically indistinguishable from the control. In addition, this treatment determines a reduction in ROS production in cells incubated with H_2_O_2_ 1 mM or 2 mM (Figure 5). In fact, ROS levels in these samples were similar to those of control cells, showing that hydrogen peroxide-induced stress is efficiently counteracted by the phenolic phytocomplex of pâté. These results indicate clearly the antioxidant capacity of the tested sample, even with higher concentration of H_2_O_2_.

### 2.5. Enzymatic Assays and PCA Analysis

The metabolism of AGS cells subjected to the above-mentioned treatments was investigated by measuring the enzymatic activity of seven key enzymes: Lactate dehydrogenase, LDH (citoplasmatic NADH re-oxidation), Glucose 6-phosphate dehydrogenase, G6PDH (pentose phosphate shunt), Enolase, ENO, Pyruvate kinase, PK (glycolysis), Citrate synthase, CS (Krebs cycle), 3-Hydroxyacyl-CoA dehydrogenase, HA-CoADH (beta oxidation) and Hexokinase, HK (glucose entrapment). These assays allowed us to evaluate the biological action of pâté separate from an enzymatic/metabolic point of view. Moreover, the possible use of the pâté as a biochemical countermeasure to oxidative stress was investigated.

**Figure 6:**
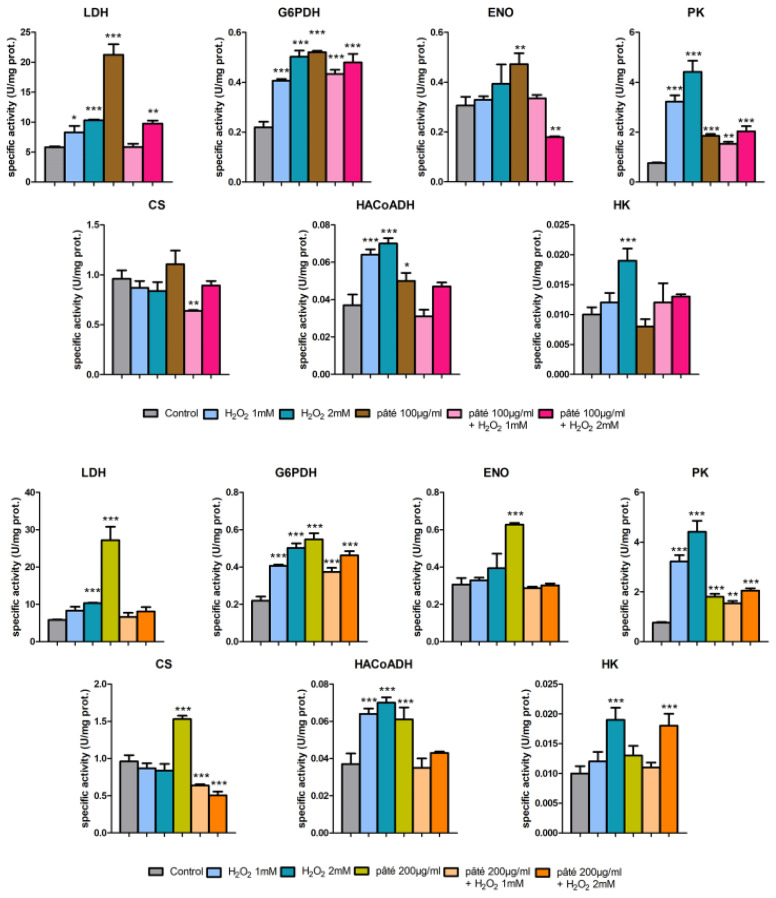
Specific activities of (from left to right and from top to bottom of each panel) LDH, G6PDH, ENO, PK, CS, HACoADH and HK for the two investigated concentrations of the extract of pâté: (**top panel**) with 100 µg/mL and (**bottom panel**) with 200 µg/mL. For each enzyme the activities were measured for control cells, cells treated with H_2_O_2_ 1 mM and 2 mM, 100 µg/mL or 200 µg/mL of the extract of pâté and cells treated with both the two concentrations of H_2_O_2_ and of the pâté, as specified in the legend. Error bars represent the standard deviation of the three replicates of the assay. Samples significantly different from control are marked with * (Student *t*-test *p* < 0.05), ** (*p* < 0.01) or *** (*p* < 0.001).

Figure 6 shows the specific activities of seven investigated enzymes in cells subjected to oxidative stress by H_2_O_2_ 1 mM and 2 mM with and without the extract of pâté pre-treatment. Each bar represents the average and standard deviation of the measurements on three replicate assays.

**Figure 7:**
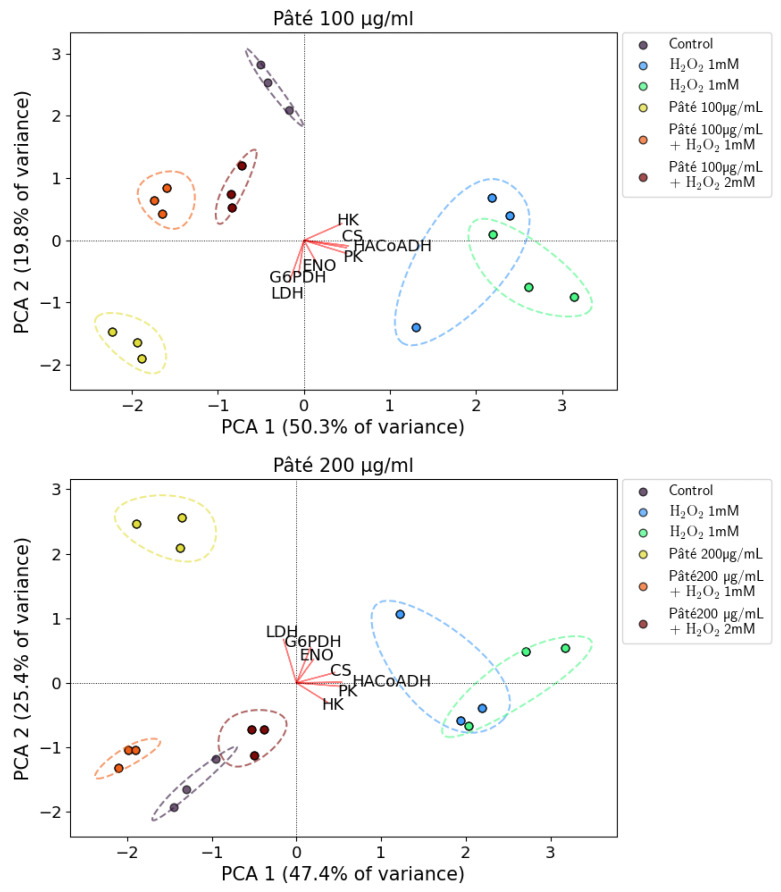
Biplots resulting from the PCA analysis for the two investigated concentrations of the extract of pâté. (**Top panel**): 100 µg/mL. (**Bottom panel**): 200 µg/mL. Coloured circles represent the various samples as specified in the legend (control cells, cells treated with H_2_O_2_ 1 mM and 2 mM, cells treated with 100 µg/mL or 200 µg/mL of the extract and cells treated with both the two concentrations of H_2_O_2_ and of the extract of pâté). The dashed lines of the same colour represent the envelope of each cluster of points to help the reader to view them. The red lines marked with the various enzymes names represent the projections of the relative enzymatic activities on the PCA1 and PCA2 coordinates.

The results of the PCA analysis of the measured enzymatic activities in the AGS cells subjected to oxidative stress by H_2_O_2_ with and without pâté as a countermeasure are shown in Figure 7. In the top and bottom panel of the figure, the results of the experiments with 100 µg/mL and 200 µg/mL of the extract of pâté are reported, respectively. In Figure 7, three points are plotted for each sample, and these points represent the average result of each of three experiments performed in triplicate.

The first two principal components explained 70.1% of the total variance (PCA1 accounting for 50.3% and PCA2 for 19.8%) for the experiments with 100 µg/mL of sample and 72.8% of the total variance (47.4% for PCA1 and 25.4% for PCA2) for the dose of the experiments with 200 µg/mL of sample.

Figure 7 highlights that the various sets of data samples are well clustered and located in distinct regions of the biplots. First of all, the clusters representing the pre-treatment alone with the two concentrations of the extract of pâté are located in a particular region of the biplots (approximately [−2; −2] and [−1.5; 2.5], respectively for 100 µg/mL and 200 µg/mL of sample). This indicates a profile of the measured enzymatic activities typical of the pâté and distinct from that of the other samples (control, H_2_O_2_ samples and H_2_O_2_ and pâté samples). This particular enzymatic profile, peculiar to the pâté treatments, can be observed also in Figure 6, where both the two concentrations of the phenolic extract of pâté produce a statistically significant increase in LDH, G6PDH, ENO, PK, HACoADH and HK activities with respect to the control. An increase in CS activity, although statistically significant only for the higher pâté concentration, was also observed. The increased activity of G6PDH and therefore the upregulation of the pentose phosphate cycle are notable, indicating that the treatment with the phenolic pool of pâté is able to make the cells ready to respond to the oxidative stress induced by H_2_O_2_.

The clusters related to the H_2_O_2_ 1 mM and 2 mM treatments are located in regions of the biplots distinct from the locations of the controls and pâté clusters (the positive and negative side of PCA1, respectively), indicating that the H_2_O_2_ treatment actually significantly changes the profile of the measured enzymatic activities. This picture confirms what was already observed in Figure 6, with the increase in the specific activities of all assayed enzymes except for CS with respect to the controls for the H_2_O_2_ 1 mM and 2 mM treatments. Moreover, it can be observed that the H_2_O_2_ 1 mM and 2 mM treatment clusters (in both top and bottom panels of Figure 7) are partially separated from each other, suggesting that the profile of the enzymatic activities depends on the H_2_O_2_ concentration. This can also be observed in Figure 6, where all the enzymatic activities, excluding that of CS, show an activity increase with the H_2_O_2_ 2 mM treatment with respect to the H_2_O_2_ 1 mM treatment.

The absence of an increase in the CS activity with H_2_O_2_ treatments observed in our results (CS activity shows a negative trend with H_2_O_2_ concentration, although not statis-tically significant) is indicative of an impaired operation of the Krebs cycle resulting from a mitochondrial alteration, due to the oxidative stress induced by H_2_O_2_, as is often reported in the literature [23]. In such a condition, the increased activities of PK (glycolysis) and HACoADH (oxidation of fatty acids) could compensate for the reduced production of mitochondrial NADH, and then ATP, by the Krebs cycle. The activity of LDH could compensate, instead, for the decrease in re-oxidation of NADH at the mitochondrial level. Therefore, this condition could require a rise in glucose consumption, confirmed by the observed increase in HK activity (glucose phosphorylation) particularly at the H_2_O_2_ 2 mM concentration [24].

Moreover, the observed increase in the activity of G6PDH with H_2_O_2_ treatments (higher for H_2_O_2_ 2 mM) is also an indication of the activation of the shunt of the pentose phosphate, of NADPH production, and therefore of the maintenance of glutathione in the reduced state—one of the main cellular defence mechanisms against oxidative stress [25].

The increased activity of G6PDH observed with H_2_O_2_ or pâté treatments could have different causes, since, unlike H_2_O_2_, pâté does not induce ROS production (Figure 5) and, in the PCA analysis, its experimental points cluster in a different quadrant with respect to H_2_O_2_.

The clusters of the samples treated with the two concentrations of the phenolic extract of pâté (for the 24 h preceding the H_2_O_2_ stimulus) and then with the two H_2_O_2_ concentrations are located in the same biplot region as the control cluster (approximately [−1; 1] and [−1; −1.5], respectively, for the sample dose of 100 µg/mL and 200 µg/mL). This indicates clearly that the cells pre-treated with the phenolic extract of pâté, in the presence of H_2_O_2_-induced oxidative stress show an enzymatic activity profile similar to that of the control cells.

Observing the results in Figure 6, this situation is evident particularly for PK and HACoADH, where the activity increases with respect to the control for the H_2_O_2_ 1 mM and 2 mM treatments and decrease approaching control levels for the H_2_O_2_+pâté samples.

These results suggest that the reduced functionality of the mitochondria and the consequent energy deficiency due to the H_2_O_2_ treatment could be counteracted by pre-treatment with the pâté. This is also suggested by the LDH activity levels.

Moreover, in the case of LDH, a significant reduction in the activity for the H_2_O_2_ 2 mM+pâté 200 µg/mL treatment can be observed with respect to H_2_O_2_ 2 mM. This activity decrease is not evident in the case of the H_2_O_2_ 2 mM+pâté 100 µg/mL treatment. This suggests that the higher concentration of the pâté, and therefore of the antioxidant phenols, is more effective at counteracting the oxidative stress condition induced by H_2_O_2_.

Evident biological activity of the pâté on AGS cells emerged from the significant increase in almost all the enzymatic activities for the samples treated with pâté with respect of control samples. In this context, pre-treatment with the phenolic extract of pâté can be interpreted as a phase in which cells could be predisposed to a biochemical state capable of counteracting subsequent oxidative stress. On the other hand, it should also be considered that pâté has, in addition to a biological role, a pure chemical role by acting directly as a scavenger of the ROS produced by H_2_O_2_ [26].

In the context of what has been observed and discussed, an important continuation of research should concern the study of how pâté allows cells to cope with oxidative stress, by acting directly as a scavenger of ROS or by modifying transcription, through the Nrf2 factor, or by modifying the activity of enzymes such as glutathione reductase, glutathione peroxidase, superoxide dismutase and also glyoxalase1 [27,28,29,30].

It will also be interesting to investigate the biological effects and metabolic activations of individual phenolic compounds, so as to clarify the possible specific role of some of the components of the phytocomplex.

## 3. Materials and Methods

### 3.1. Phenolic Extract of Pâté

The extraction method was that described by Cecchi et al. (2018), with slight modification as briefly summarized below. A quantity of 2.5 g of lyophilized pâté was extracted twice with 50 mL of EtOH:H_2_O 80:20 *v*/*v*, under magnetic stirring for 1 h each time, then centrifuged for 10 min at 10 °C and 1667× *g*, after which the supernatant was collected. The solutions of the first and second steps were pooled and defatted with *n*-hexane (20 mL × 3) and liquid/liquid extraction. The hexane was discarded and the solution was dried under a vacuum with a rotavapor obtaining 856 mg. Part of the sample was divided into vials, dried and stored in a freezer until bioassays. One aliquot was used for the HPLC analysis and acid hydrolysis according to a previous work [31]. The acidic hydrolysis of the phenolic extract was carried out by first extracting 300 μL of the sample added with 300 μL of 1 M H_2_SO_4_. The sample was then placed in oven at 80 °C for 2 h. After cooling at room temperature, 400 μL of distilled water was added and the solution was centrifuged and analysed using HPLC-DAD to determine the total content of tyrosol and hydroxytyrosol (free and linked forms).

### 3.2. Analyses by HPLC-DAD-MS of the Phenolic Extract from Pâté

The HPLC-DAD analyses were carried out using a HP1100 liquid chromatograph, provided with autosampler, column heater module and DAD detector (Agilent, Palo Alto, CA, USA). The column was a Poroshell 120, EC-C18 (150 × 3.0 mm, 2.7 µm ps; Agilent, Palo Alto, CA, USA); the analyses were performed at 26 °C; the mobile phase (flow 0.4 mL/min) was constituted by CH_3_CN (A) and H_2_O at pH 3.2 by HCOOH (B). The applied linear gradient was as follows: Solvent B ranged from 95% to 60% in 40 min, with a plateau of 5 min to 60%, then 5 min to reach 0% B and a final plateau of 3 min; the last 2 min was the return to 95% B. Post-run equilibration time was 10 min and injection volumes 2 μL for the sample before hydrolysis and 20 μL after hydrolysis. Chromatograms were recorded at 280 nm. LC-MS analysis was performed using HP 1260 MSD coupled with DAD and MSD detectors, and an API/electrospray interface (all from Agilent Technologies). The HPLC method was the same as previously described. For the MS detector, the following settings were used: capillary voltage 3500 V; drying gas flow 12.0 L/min, temperature 350 °C; and nebulizer pressure 1811 Torr. The acquisition was carried out in full spectrum scan (range 100–1200 Th) in negative ionization (fragmentor at 150–200 V). The quantitation of phenolic compounds was performed at 280 nm using several pure standards, as reported below. The curve of tyrosol (linearity range 0–1.21 µg and R^2^ = 0.9999) was used to express tyrosol, hydroxytyrosol and their glucosides. The calibration curve of oleuropein (0–3.16 µg and R^2^ = 0.9986) was used to evaluate secoiridoid derivatives and total phenolic content. The calibration curve of luteolin-7-*O*-glucoside (0–1.57 µg and R^2^ = 0.9956) was used for luteolin, rutin and luteolin-7-*O*-glucoside. Verbascoside and β-OH acteoside isomers 1 and 2 were quantified with the calibration curve of verbascoside (0–1.96 µg and R^2^ = 0.9996), and the curve of caffeic acid (0–0.96 µg and R^2^ = 0.9989) was applied for chlorogenic acid and cafselogoside. The total phenolic content was calculated as the sum of each phenolic compound. Previously determined correction factors were applied for the calculation of hydroxytyrosol and verbascoside [32].

### 3.3. Cell Line

AGS cells (ATCC CRL-1739) were purchased from Sigma-Aldrich (MERCK, Darmstadt, Germany) [33]. They are derived from a gastric adenocarcinoma and are commonly used as a model of gastric mucosal cells. The cells were grown in F-12 K medium (Hyclone, GE Healthcare Lifesciences, Marlborough, MA, USA) with 10% foetal bovine serum (FBS, Biowest, Nuaillé, France) at 37 °C in a 5% CO_2_ humidified atmosphere. Cells (1 × 10^6^) were seeded in 10 cm Petri dishes and propagated every 2 days by detaching with a 0.25% trypsin/EDTA solution from Sigma-Aldrich (MERCK, Darmstadt, Germany). Cultures were periodically tested for *Mycoplasma* spp. contamination.

### 3.4. AGS Cells Viability (MTT Test)

For the assessment of the effects on viability of the pâté, the AGS cells were seeded in a 96-multiwell plate at a density of 12,000 cells/well, and after 48 h were incubated for 2, 24 and 48 h with 50, 100 and 200 μg/mL of the extract of pâté reconstituted with Dimethyl sulfoxide (DMSO). Control samples were incubated with DMSO only.

At the end of incubation, cell viability was measured by incubating with 1 mM thiazolyl blue tetrazolium bromide (MERCK, Darmstadt, Germany) in culture medium for 40 min in the dark (MTT test). Then, the medium was removed and DMSO was added to dissolve formazan crystals. The absorbance signal at 570 nm was read on the multiplate reader (Infinite M200PRO, Tecan, Mannedorf, Switzerland) and the background absorbance at 630 nm was subtracted from signal absorbance to obtain normalized absorbance values.

### 3.5. Cytofluorimetric Analysis of Cell Cycle

To analyse the AGS cell distribution in the cell cycle phases after incubation with pâté at different concentrations and times, 250,000 cells were seeded in 6 cm Petri dishes and after 48 h incubated with pâté at 50, 100 and 200 μg/mL. At the end of incubation, the cells were collected by trypsinization and stained with propidium iodide according to the method of [34]. Samples were analysed using a FACScan flow cytometer (Becton Dickinson, Milan, Italy). Quantification of cells in the different cycle phases was performed by ModFit LT software, version 3.0 (Verity Software House Inc., Topsham, ME, USA).

### 3.6. Scratch Assay

AGS cells were seeded (2.5 × 10^5^) on 6 cm Petri dishes and cultured until ∼80% of confluence. Then, monolayers were gently scratched with a 200 μL pipette tip. Culture medium and detached cells were removed and monolayers were washed twice with phosphate-buffered solution (PBS). Fresh complete medium with 100 μg/mL of pâté was added to cells (in control samples, no fresh pâté was added to the complete medium). Samples where then observed at the microscope as detailed in the following subsection; this moment is considered 0 delay time of the scratch closure to which we refer in the following.

Experiments were performed realizing twin scratch assays with the role of control and treatment. Each control–treatment experiment was performed in triplicate.

Scratch assay imaging was performed with an inverted microscope in phase contrast configuration (Leica DM IL, Leica Microsystems GmbH, Wetzlar, Germany) equipped with a 5x objective and a Canon CCD camera (Canon Power shot S40, 2272 × 1074, ~23 × 20 μm pixels, Canon Inc., Tokyo, Japan), obtaining a field of view of ~2.5 × 2.5 mm necessary to observe the entire scratch width (~0.6–1.4 mm). Images of the scratch were acquired approximately once an hour (except for the unavoidable night-time gap) from the starting time to ∼25–40 h, thus guaranteeing 10–12 images.

Dedicated image analysis routines written in the Python language (Python Software Foundation, version 3.8, available at https://www.python.org/) allowed measuring the scratch width for each image. Then, for each sample and delay time, mean scratch width and standard deviation on the triplicate was calculated. Finally, the scratch width vs. delay time curve for each sample was constructed.

This curve was then modelled by means of least squares minimization with a linear model calculating the scratch closure velocity and closure-time (i.e., the time at which the scratch is completely closed according to the linear model).

For any detail regarding image analysis and scratch closure modelling, refer to [35].

### 3.7. Intracellular ROS Production Assay

The level of intracellular ROS was quantified using fluorescence with 2′,7′-dichlorofluorescein diacetate (DCF-DA, MERCK, Darmstadt, Germany) as described by [36]. Cells were plated in a 96-multi-well (18,000 cells/well), and after 24 h were incubated with 100 or 200 µg/mL of pâté and 25 μM DCF-DA (dissolved in culture medium) for a day. Then, the cells were treated with H_2_O_2_, 1 or 2 mM, for 1 h (chosen concentration which determines a detectable ROS production in AGS cells, (data with lower H_2_O_2_ concentration not shown). Immediately after the end of treatment with H_2_O_2_, samples were washed twice with PBS; the relative levels of fluorescence emission were quantified in the multiplate reader (excitation: 485 nm, emission: 535 nm).

### 3.8. Enzymatic Assays

For the determination of the activities of the assayed enzymes, representative of the principal metabolic pathways, 4 × 10^5^ cells were seeded in 10 cm Petri dishes. After one day, 24 h incubation began with 100 and 200 µg/mL of pâté, and then the cells were treated with H_2_O_2_ 1 or 2 mM for 1 h. At the end of the treatment the cells were collected by trypsinization.

Cells were quickly rinsed in ice-cold phosphate-buffered saline (PBS, 10 mM sodium phosphate and 0.15 M NaCl, pH 7.2) and frozen. At the time of use, after thawing the sample at room temperature and 30 min of incubation on ice, cells were lysed by sonication (three short bursts) at 4 °C in 50 mM Tris, pH 7.4, containing 5 mM dithiothreitol and Sigma protease inhibitors mix (1/100, *v*/*v*) and centrifuged at 12,000× *g* in a microcentrifuge at 4 °C for 30 min.

Total protein content [37] and enzymatic activities of the assayed enzymes were quantified in supernatants.

The determinations of the enzymatic activities of Pyruvate Kinase (PK, EC 2.7.1.40), Enolase (ENO, EC 4.2.1.11), Glucose-6-P dehydrogenase (G6PDH, EC 1.1.1.49), Lactate dehydrogenase (LDH, EC 1.1.1.27), Hexokinase (HK, EC 2.7.1.1), 3-Hydroxyacyl-CoA dehydrogenase (HACoADH, EC 1.1.1.35) and Citrate synthase (CS, EC 4.1.3.7) were performed at 37 °C, according to Bergmeyer ([38], pp. 443–444, 449, 458–459, 481–482, 473–474, 509–510), with slight modifications, continuously following NADH or NADPH appearance/disappearance at 340 nm, using an UV-2100 spectrophotometer (Shimadzu, Columbia, MD, USA).

All the enzymatic reactions were started by adding the substrate.

One unit of activity is defined as the quantity of enzyme which transforms 1 μmole of substrate in 1 min. The value of 6.22 mM^−1^ cm^–1^ is considered to be the NADH (or NADPH) molar extinction co-efficient.

All the reagents used for enzymatic assays were purchased from Merk (MERCK, Darmstadt, Germany).

### 3.9. Principal Component Analysis (PCA) of Enzymatic Activities

To discuss how the investigated treatments influence the global profile of the measured seven enzymatic activities for the AGS cells, PCA analysis was performed [39]. In each of the three experiments, the following samples were prepared in triplicate: control, H_2_O_2_ 1 mM, H_2_O_2_ 2 mM, extract of pâté 100 µg/mL; extract of pâté 200 µg/mL, H_2_O_2_ 1 mM with 100 µg/mL extract of pâté, H_2_O_2_ 2 mM with 100 µg/mL extract of pâté, H_2_O_2_ 2 mM with 100 µg/mL extract of pâté and H_2_O_2_ 2 mM with 200 µg/mL extract of pâté. Each PCA data point represents the average of that sample on one experiment. The principal components for this dataset were calculated and the results were plotted in the form of biplots showing the two principal components accounting for the highest fraction of the original data variance. PCA analysis was performed separately for the two concentrations of pâté: because the decomposition technique based on the data variance, inserting a unique dataset into the two investigated pâté concentrations could increase the data variance and lead to an incorrect interpretation of the results. Therefore, PCA was performed independently on a dataset relative to the dose of 100 µg/mL extract of pâté and on another dataset relative to the dose of 200 µg/mL extract of pâté.

### 3.10. Statistical Analysis

All the experiments were carried out in triplicate. The differences between treated and control samples observed in biological activity assessment assays such as the MTT Test, ROS Production Assay, flow cytometry and enzymatic activity, were analysed using Student’s t-test (significance level of 0.05). Furthermore, to evaluate the statistical significance of the trend over time of MTT Test and enzymatic activities assay, a two-way analysis of variance was carried out (ANOVA test with significance level on the interaction parameter of 0.05). ANOVA and Student’s *t*-test were performed using GraphPad Prism version 5.03 (GraphPad Software, San Diego, CA, USA, www.graphpad.com).

## 4. Conclusions

The main results of this study, which evaluated the effects of a phenolic phytocomplex derived from virgin olive oil production on an AGS cell line for the first time, were not only the absence of toxicity at the investigated concentrations, and the reduction in the ROS produced by an oxidative stress, but also the strong impact on some central metabolic pathways. It can be supposed that different mechanisms are behind the observed effects, and certainly, both the biological and chemical actions of the phenolic extract of pâté contribute to the observed results. The pre-treatment with the pool of antioxidant compounds of pâté helps the cells to efficaciously counteract a general oxidative stress induced by hydrogen peroxide both restoring the basal level of ROS and modulating the activities of the investigated enzymes. This latter part of the study is an innovative aspect never investigated before for AGS cells treated with natural phenolic molecules.

Future research will concern the study of how pâté allows cells to counteract the oxidative stress, either as an ROS scavenger or by modifying transcription/activity of enzymes involved in ROS detoxification. Moreover, it will also be interesting to investigate the role of individual components of the phytocomplex in terms of biological effects and metabolic activations.

## Figures and Tables

**Table 1:** Closure velocity and closure time of the scratch after treatment with the hydroalcoholic extract of pâté.

	Scratch Closure Velocity [μm/h]	Scratch Closure Time [h]
Control	11.1 ± 1.7	46.0 ± 7.8
pâté	9.2 ± 1.0	50.6 ± 5.8

## Data Availability

Not applicable.

## References

[1] Galanakis C.M. (2012). Recovery of high added-value components from food wastes: Conventional, emerging technologies and commercialized applications. Trends Food Sci. Technol..

[2] Romani A., Ieri F., Urciuoli S., Noce A., Marrone G., Nediani C., Bernini R. (2019). Health effects of phenolic compounds found in extra-virgin olive oil, by-products, and leaf of *Olea europaea* L.. Nutrients.

[3] EFSA Panel on Dietetic Products, Nutrition and Allergies (NDA) (2011). Scientific Opinion on the Substantiation of Health Claims Related to Polyphenols in Olive Oil and Protection of LDL Particles from Oxidative Damage. EFSA J..

[4] Cecchi L., Migliorini M., Zanoni B., Breschi C., Mulinacci N. (2018). An effective HPLC-based approach for the evaluation of the content of total phenolic compounds transferred from olives to virgin olive oil during the olive milling process. J. Sci. Food Agric..

[5] Cecchi L., Migliorini M., Giambanelli E., Canuti V., Bellumori M., Mulinacci N., Zanoni B. (2022). Exploitation of virgin olive oil by-products (*Olea europaea* L.): Phenolic and volatile compounds transformations phenomena in fresh two-phase olive pomace (‘alperujo’) under different storage conditions. J. Sci. Food Agric..

[6] Cecchi L., Bellumori M., Cipriani C., Mocali A., Mulinacci N., Giovannelli L. (2018). A two-phase olive mill by-product (pâté) as a convenient source of phenolic compounds: Content, stability, and antiaging properties in cultured human fibroblasts. J. Funct. Foods.

[7] Mulinacci N., Valletta A., Pasqualetti V., Innocenti M., Giuliani C., Bellumori M., De Angelis G., Carnevale A., Locato V., Di Venanzio C. (2019). Effects of ionizing radiation on bioactive plant extracts useful for preventing oxidative damages. Nat. Prod. Res..

[8] Bellumori M., De Marchi L., Mainente F., Zanoni F., Cecchi L., Innocenti M., Mulinacci N., Zoccatelli G. (2021). A by-product from virgin olive oil production (pâté) encapsulated by fluid bed coating: Evaluation of the phenolic profile after shelf-life test and in vitro gastrointestinal digestion. Int. J. Food Sci. Technol..

[9] Vitali Čepo D., Radić K., Jurmanović S., Jug M., Grdić Rajković M., Pedisić S., Moslavac T., Albahari P. (2018). Valorization of Olive Pomace-Based Nutraceuticals as Antioxidants in Chemical, Food, and Biological Models. Molecules.

[10] Giuliani C., Marzorati M., Daghio M., Franzetti A., Innocenti M., de Wiele T.V., Mulinacci N. (2019). Effects of olive and pomegranate by-products on human microbiota: A study using the SHIME^®^ in vitro simulator. Molecules.

[11] Parisio C., Lucarini E., Micheli L., Toti A., Bellumori M., Cecchi L., Calosi L., Bani D., Di Cesare Mannelli L., Mulinacci N. (2020). Extra virgin olive oil and related by-products (*Olea europaea* L.) as natural sources of phenolic compounds for abdominal pain relief in gastrointestinal disorders in rats. Food Funct..

[12] Balli D., Cecchi L., Innocenti M., Bellumori M., Mulinacci N. (2021). Food by-products valorisation: Grape pomace and olive pomace (pâté) as sources of phenolic compounds and fiber for enrichment of tagliatelle pasta. Food Chem..

[13] Cecchi L., Schuster N., Flynn D., Bechtel R., Bellumori M., Innocenti M., Mulinacci N., Guinard J.X. (2019). Sensory profiling and consumer acceptance of pasta, bread and granola bar fortified with dried olive pomace (pâté)—A by-product from virgin olive oil production. J. Food Sci..

[14] Terramoccia S., Bartocci S., Taticchi A., Di Giovanni S., Pauselli M., Mourvaki E., Urbani S., Servili M. (2013). Use of dried stoned olive pomace in the feeding of lactating buffaloes: Effect on the quantity and quality of the milk produced. Asian-Australas. J. Anim. Sci..

[15] Dinu M., Pagliai G., Scavone F., Bellumori M., Cecchi L., Nediani C., Maggini N., Sofi F., Giovannelli L., Mulinacci N. (2021). Effects of an Olive By-Product Called Pâté on Cardiovascular Risk Factors. J. Am. Coll. Nutr..

[16] Faraoni P., Gnerucci A., Ranaldi F., Orsini B., Romano G., Fusi F. (2018). Side effects of intra-gastric photodynamic therapy: An in vitro study. J. Photochem. Photobiol. B.

[17] Halliwell B., Rafter J., Jenner A. (2005). Health promotion by flavonoids, tocopherols, tocotrienols, and other phenols: Direct or indirect effects? Antioxidant or not?. Am. J. Clin. Nutr..

[18] Speciale A., Anwar S., Canali R., Chirafisi J., Saija A., Virgili F., Cimino F. (2013). Cyanidin-3-O-glucoside counters the response to TNF-alpha of endothelial cells by activating Nrf2 pathway. Mol. Nutr. Food Res..

[19] Cheli F., Baldi A. (2011). Nutrition-based health: Cell-based bioassays for food antioxidant activity evaluation. J. Food Sci..

[20] Xu B., Chang S.K. (2010). Phenolic substance characterization and chemical and cell-based antioxidant activities of 11 lentils grown in the northern United States. J. Agric. Food Chem..

[21] Theoduloz C., Burgos-Edwards A., Schmeda-Hirschmannb G., Jiménez-Aspee F. (2018). Effect of polyphenols from wild Chilean currants (*Ribes* spp.) on the activity of intracellular antioxidant enzymes in human gastric AGS cells. Food Biosci..

[22] Ávila F., Theoduloz C., López-Alarcón C., Dorta E., Schmeda-Hirschmann G. (2017). Cytoprotective Mechanisms Mediated by Polyphenols from Chilean Native Berries against Free Radical-Induced Damage on AGS Cells. Oxid. Med. Cell. Longev..

[23] Rusz M., Del Favero G., El Abiead Y., Gerner C., Keppler B.K., Jakupec M.A., Koellensperger G. (2021). Morpho-metabotyping the oxidative stress response. Sci. Rep..

[24] Molavian H.R., Kohandel M., Sivaloganathan S. (2016). High Concentrations of H_2_O_2_ Make Aerobic Glycolysis Energetically More Favorable for Cellular Respiration. Front. Physiol..

[25] Grant C.M. (2008). Metabolic reconfiguration is a regulated response to oxidative stress. J. Biol..

[26] Hollman P.C., Cassidy A., Comte B., Heinonen M., Richelle M., Richling E., Serafini M., Scalbert A., Sies H., Vidry S. (2011). The biological relevance of direct antioxidant effects of polyphenols for cardiovascular health in humans is not established. J. Nutr..

[27] Thakur V.S., Gupta K., Gupta S. (2012). The chemopreventive and chemotherapeutic potentials of tea polyphenols. Curr. Pharm. Biotechnol..

[28] Son T.G., Camandola S., Mattson M.P. (2008). Hormetic dietary phytochemicals. Neuromol. Med..

[29] Orena S., Owen J., Jin F., Fabian M., Gillitt N.D., Zeisel S.H. (2015). Extracts of Fruits and Vegetables Activate the Antioxidant Response Element in IMR-32 Cells. J. Nutr..

[30] Vriend J., Reiter R.J. (2015). The Keap1-Nrf2-antioxidant response element pathway: A review of its regulation by melatonin and the proteasome. Mol. Cell. Endocrinol..

[31] Cecchi L., Khatib M., Bellumori M., Civa V., Domizio P., Innocenti M., Balli D., Mulinacci N. (2023). Industrial drying for agrifood by-products re-use: Cases studies on pomegranate peel (*Punica granatum* L.) and stoned olive pomace (pâtè, *Olea europaea* L.). Food Chem..

[32] Bellumori M., Cecchi L., Romani A., Mulinacci N., Innocenti M. (2018). Recovery and stability over time of phenolic fractions by an industrial filtration system of olive mill wastewaters: A three-year study. J. Sci. Food Agric..

[33] Barranco S.C., Townsend C.M., Casartelli C., Macik B.G., Burger N.L., Boerwinkle W.R., Gourley W.K. (1983). Establishment and characterization of an in vitro model system for human adenocarcinoma of the stomach. Cancer Res..

[34] Vindeløv L.L., Christensen I.J. (1990). A review of techniques and results obtained in one laboratory by an integrated system of methods designed for routine clinical flow cytometric DNA analysis. Cytometry.

[35] Gnerucci A., Faraoni P., Sereni E., Ranaldi F. (2020). Scratch assay microscopy: A reaction-diffusion equation approach for common instruments and data. Math. Biosci..

[36] Bass D.A., Parce J.W., Dechatelet L.R., Szejda P., Seeds M.C., Thomas M. (1983). Flow cytometric studies of oxidative product formation by neutrophils: A graded response to membrane stimulation. J. Immunol..

[37] Bradford M.M. (1976). A rapid and sensitive method for the quantitation of microgram quantities of protein utilizing the principle of protein-dye binding. Anal. Biochem..

[38] Bergmeyer H.U. (1974). Methods of Enzymatic Analysis.

[39] Jolliffe I.T., Cadima J. (2016). Principal component analysis: A review and recent developments. Philos. Trans. A Math. Phys. Eng. Sci..

